# Gene Expression and Stress Response Mediated by the Epigenetic Regulation of a Transposable Element Small RNA

**DOI:** 10.1371/journal.pgen.1002474

**Published:** 2012-02-09

**Authors:** Andrea D. McCue, Saivageethi Nuthikattu, Sarah H. Reeder, R. Keith Slotkin

**Affiliations:** 1 Department of Molecular Genetics, The Ohio State University, Columbus, Ohio, United States of America; 2 The Center for RNA Biology, The Ohio State University, Columbus, Ohio, United States of America; National Institute of Genetics, Japan

## Abstract

The epigenetic activity of transposable elements (TEs) can influence the regulation of genes; though, this regulation is confined to the genes, promoters, and enhancers that neighbor the TE. This local *cis* regulation of genes therefore limits the influence of the TE's epigenetic regulation on the genome. TE activity is suppressed by small RNAs, which also inhibit viruses and regulate the expression of genes. The production of TE heterochromatin-associated endogenous small interfering RNAs (siRNAs) in the reference plant *Arabidopsis thaliana* is mechanistically distinct from gene-regulating small RNAs, such as microRNAs or *trans*-acting siRNAs (tasiRNAs). Previous research identified a TE small RNA that potentially regulates the *UBP1b* mRNA, which encodes an RNA–binding protein involved in stress granule formation. We demonstrate that this siRNA, siRNA854, is under the same *trans*-generational epigenetic control as the *Athila* family LTR retrotransposons from which it is produced. The epigenetic activation of *Athila* elements results in a shift in small RNA processing pathways, and new 21–22 nucleotide versions of *Athila* siRNAs are produced by protein components normally not responsible for processing TE siRNAs. This processing results in siRNA854's incorporation into ARGONAUTE1 protein complexes in a similar fashion to gene-regulating tasiRNAs. We have used reporter transgenes to demonstrate that the *UPB1b* 3′ untranslated region directly responds to the epigenetic status of *Athila* TEs and the accumulation of siRNA854. The regulation of the *UPB1b* 3′ untranslated region occurs both on the post-transcriptional and translational levels when *Athila* TEs are epigenetically activated, and this regulation results in the phenocopy of the *ubp1b* mutant stress-sensitive phenotype. This demonstrates that a TE's epigenetic activity can modulate the host organism's stress response. In addition, the ability of this TE siRNA to regulate a gene's expression in *trans* blurs the lines between TE and gene-regulating small RNAs.

## Author Summary

The portion of the genome that does not encode for genes is often overlooked as a source of cellular regulatory information. Here, we demonstrate that regulatory information controlling expression and protein production from a gene called *UBP1b* is coming from a distant non-gene transposable element (TE). TEs are fragments of DNA that, unlike genes, are capable of duplicating themselves from one location in the genome to another, and occupy nearly half of the human genome. TEs are often referred to as “junk DNA,” as the study of cellular regulation and function is focused on genes. The regulation of TEs is distinct from genes, as a process termed *epigenetic silencing* heritably represses TE expression and activity. We have demonstrated that the epigenetic status (active versus silenced) of the *Athila* TE family regulates the *UBP1b* gene through the activity of a TE small RNA. The function of the *UPB1b* gene is to respond to and regulate cellular stress, and the epigenetic regulatory status of the *Athila* TE therefore modulates this stress response. This demonstrates that the epigenetic regulation of TEs can be a source of gene regulatory information, influencing a basic cellular function such as the stress response.

## Introduction

Transposable elements (TEs) are mobile fragments of DNA that can accumulate and occupy large fractions of a genome, including over 45% of the human genome [1]. When active, TEs have the potential to create mutations by inserting into genes or generating breaks in DNA. To suppress the mutagenic potential of TEs, the eukaryotic genome has evolved defense mechanisms to inhibit TE proliferation, which are distinct from the developmental regulation of genes [2]. TEs are targeted for epigenetic repression mediated by the overlapping signals of cytosine DNA methylation, repressive histone tail modifications, and remodeling of chromatin into transcriptionally recalcitrant condensed heterochromatin (reviewed in [3]). Gene regulation can be influenced by the epigenetic regulation of TEs; however, this only occurs due to the proximity of a preexisting or newly transposed TE to a gene. This regulation of genes by neighboring TEs in *cis* can be due to multiple mechanisms, including interruption of a regulatory element, or by local spreading of repressive chromatin modifications such as DNA or histone methylation, resulting in position-effect variegation and potentially the formation of heritable epialleles [4]–[5].

TEs are major producers of small RNAs that act to maintain the TE in an epigenetically silenced state. In plants, and perhaps in animals, heterochromatin modifications are targeted by the activity of small RNAs. For example, in the mouse TE-derived *piwi*-interacting RNAs (piRNAs) guide DNA methylation to TEs [6]. In the reference plant *Arabidopsis thaliana*, the cycle of RNA-directed DNA methylation (RdDM) is initiated by the plant-specific *RNA Polymerase IV* (*PolIV*), which produces a non-protein coding transcript that is converted into double stranded RNA (dsRNA) by the activity of *RNA-dependent RNA Polymerase 2* (*RDR2*)(reviewed in [7]). *Dicer-like 3* (*DCL3*) cleaves this TE dsRNA into small interfering RNAs (siRNAs) of 24 nucleotides (nt) in length, which are incorporated into either *Argonaute 4* (*AGO4*), *AGO6*, or potentially *AGO9* [8]. These siRNA-loaded *Argonaute* proteins act to maintain the heterochromatic state of TEs by targeting them for DNA and histone tail methylation.

*Athila* LTR retrotransposons are the largest family of TEs in *Arabidopsis*, occupying over 2.7% of the genome [9]. *Athila* elements are transcriptionally silenced, and silencing is dependent on symmetrical CG DNA methylation. When DNA methylation is removed, either in a DNMT1-homolog *maintenance of DNA methylation 1* (*met1*) mutant, or in a *swi/snf* family chromatin remodeling protein *decrease in DNA methylation 1* (*ddm1*) mutant, transcriptional activation occurs [10]–[11]. *Athila* retrotransposons are also transcriptionally activated in the vegetative nucleus of wild-type (wt) pollen grains [12]. In all of these examples heterochromatin modifications and condensation are lost, and global activation of TEs occurs [4], [12]–[13].

Upon global activation of TEs, there are widespread shifts in the accumulation of small RNAs derived from TE transcripts. Transcriptional activation of most silenced TEs results in the loss of their corresponding siRNAs [12]–[14]. However, some retrotransposon families, including *Athila*, are unusual in the fact that they produce more siRNAs when epigenetically active than when epigenetically silenced [12], [15]–[16]. The *Athila* siRNAs that increase in abundance are primarily 21–22 nt in length and are produced from the non-protein coding region downstream of the *gag* and *pol* ORFs of the consensus *Athila* element. The abundance and specific location of these siRNAs generates islands of 21–22 nt siRNAs in the genome when epigenetic silencing of *Athila* is lost [12].

In *Arabidopsis*, as well as in animals, the production of small RNAs and subsequent targeting of TEs is distinct from the production of gene-regulating small RNAs (reviewed in [17]). The first examples of a TE piRNA or siRNA regulating a genic mRNA in *trans* were only recently discovered in *Drosophila* and mouse [18]–[19]. In addition, recently an example of a plant viral siRNA was shown to regulate a gene [20]. However, these examples represent exceptions to the general rule of separation between TE/viral and gene-regulating small RNAs [21]. For example, there is a clear distinction between the biogenesis mechanism and target of TE siRNAs and microRNAs. MicroRNAs act in plants and animals to regulate gene expression on the post-transcriptional or translational level. In *Arabidopsis*, DCL1 produces 21 nt microRNAs from stem-loop precursor transcripts generated by RNA polymerase II (PolII), and these microRNAs are loaded primarily into AGO1. Thus, microRNAs are not amplified by an RNA-dependent RNA polymerase, and only one or two single small RNA species accumulate from the microRNA locus. In contrast, most plant siRNAs are the products of RNA-dependent RNA polymerases, and cleavage of these long dsRNAs produces clusters of siRNAs from each locus. However, the notion that only microRNAs regulate genes, while endogenous siRNAs do not, is incorrect, as some inverted repeat-derived siRNAs act to regulate genes, [22] and other siRNAs act to regulate genes through the *trans*-acting siRNA (tasiRNA) pathway in *Arabidopsis*. This pathway begins with the cleavage of a non-protein coding transcript by the microRNA-loaded AGO1 or AGO7, which initiates the DCL4- and RDR6-dependent phased production of siRNAs (reviewed in [23]). These siRNAs are loaded into AGO1 and regulate gene expression similar to a microRNA. DCL4, RDR6 and AGO1, as well as DCL2, also act on viral transcripts in the virus-induced gene silencing (VIGS) pathway to initiate and amplify the 21–22 nt siRNA signal that participates in the post-transcriptional degradation of the viral mRNAs, as well as to transport these siRNAs to unaffected regions of the plant to mount a systemic resistance to the spread of the virus [24]–[28]. Therefore, the *Arabidopsis* AGO1 protein is highly versatile, as it is involved in the microRNA, tasiRNA and VIGS pathways. It is currently unknown if, how or why AGO1 distinguishes between a gene-regulating tasiRNA and a VIGS siRNA involved in viral transcript processing, as both are generated using the same DCL4 and RDR6 machinery.

Arteaga-Vázquez *et al* demonstrated that 12 elements of the *Athila6* subfamily each encode a small RNA, for which they predicted and provided indirect evidence targets a genic transcript for translational repression [29]. They predicted that this small RNA was generated as a microRNA from a stem-loop precursor transcript and determined that it was processed by the microRNA machinery DCL1, HEN1 and HYL1. Additionally, they predicted that this microRNA, which they named microRNA854, targets the 3′ untranslated region (UTR) of the *UPB1b* gene, a homolog of the mammalian TIA-1 that encodes an RNA binding protein involved in the formation of stress granules [30]–[31]. They observed that 21 nt microRNA854 accumulates in wt vegetative tissues and found that the microRNA854-targeted *UPB1b* 3′UTR inhibits translation in wt plants when the 3′UTR is added to a reporter transcript. Lastly, Arteaga-Vázquez *et al* provided evidence that microRNA854 is highly conserved from plants to mammals.

We were unable to detect the accumulation of 21 nt microRNA854 in wt seedling, root, leaf and inflorescence tissues. Due to the failure to meet multiple criteria in order to validate this small RNA as a microRNA [32], including the biogenesis pathway of this small RNA (see below), we have renamed this small RNA siRNA854 to avoid confusion. We have directly demonstrated that the TE-derived siRNA854 regulates in *trans* the transcript of the *UBP1b* gene. We show that the accumulation of siRNA854 is under the same *trans*-generational epigenetic regulation and inheritance patterns to which *Athila* TEs are subject. Upon *Athila6* epigenetic activation, siRNA854 production is shifted from a 24 nt TE siRNA dependent on *PolIV* and *RDR2*, to 21–22 nt siRNAs that are dependent upon *DCL2*, *DCL4* and *RDR6* and are incorporated into AGO1. We demonstrate that *UBP1b* regulation is altered only when *Athila6* is epigenetically activated, resulting in the phenocopy of the stress-sensitive *upb1b* mutant phenotype.

## Results

### Epigenetic activation of *Athila6* results in production of *Athila* siRNAs and siRNA854

To determine when siRNA854 accumulates, we interrogated publicly available deep sequencing small RNA libraries [33] and found that in the plant body of wild-type Columbia ecotype plants (wt Col), 21 nt siRNA854 does not accumulate. Only one read of 21 nt siRNA854 was detected in over 6.6 million genome-matched reads of wt Col leaf and inflorescence small RNAs combined (Table 1). However, when epigenetic repression of *Athila6* is lost, 21 nt siRNA854 levels increase. Table 1 shows that, compared to the extremely low levels in wt Col inflorescence and leaf tissue, siRNA854 accumulates in *met1* and *ddm1* mutant inflorescences. Increased siRNA854 levels were also detected in pollen of wt Col plants, albeit to a lower level of 21 nt siRNA854 reads per million than in *met1* or *ddm1* mutants.

**Table 1:** Frequency of 21 nt siRNA854 in SBS small RNA libraries.

	siRNA854 raw counts	genome matched reads^A^	siRNA854 N 1M^B^	Reference
Col inflorescence	0	2,516,337	0	[13]
*met1* inflorescence	290	1,506,711	192	[13]
Col inflorescence	1	3,200,398	1	[12]
*ddm1* inflorescence	268	3,568,226	75	[12]
Col leaf	0	926,951	0	[16]
Col pollen	14	551,394	25	[12]

A Does not include tRNA, rRNA, snRNA, snoRNA reads.

B Number of siRNA854 reads normalized per 1 million.

**Figure 1:**
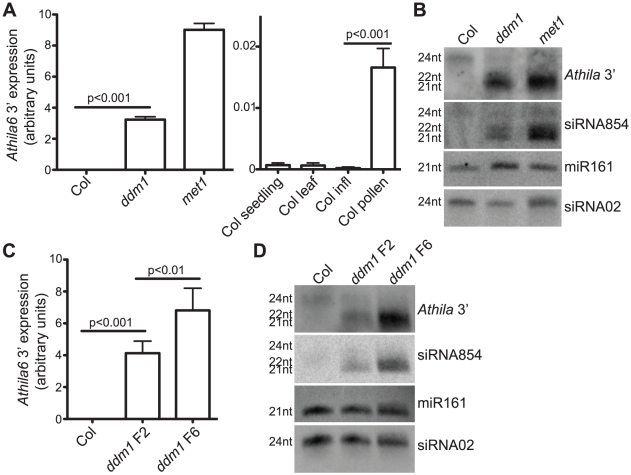
Expression of the *Athila6* retrotransposon leads to accumulation of *Athila* 21–22 nt siRNAs, including siRNA854. (A) qRT-PCR of the *Athila6* retrotransposon, using primers specific for a location 3′ of the *gag/pol* protein coding region. Expression in wt Col inflorescences (infl) is activated in *ddm1* and *met1* mutants (left). Expression is also significantly activated (although to a lesser degree) in Col pollen compared to Col whole seedling, leaf and infl. *Athila6* transcript accumulation increases >80-fold in wt pollen compared to wt Col infl. *Athila6* transcript accumulation increases >24,000-fold in *ddm1* mutants compared to wt Col infl expression. (B) Northern blot detecting siRNA854 and the flanking 3′ region of *Athila6* (*Athila6* 3′) in *ddm1* and *met1* mutant infl. 21–22 nt *Athila6* siRNAs and siRNA854 only accumulate when the retrotransposon is transcriptionally activated. The DNA oligonucleotide probe used to detect siRNA854 is 21 nt in length, and is shown in Table S1. (C) qRT-PCR of the *Athila6* retrotransposon region 3′ of *gag/pol*, demonstrating that higher retrotransposon expression levels accumulate when *ddm1* is maintained as a homozygote over several generations. (D) Small RNA Northern blot detecting siRNA854 and the *Athila6* 3′ region. Increased levels of *Athila6* 21–22 nt siRNAs and siRNA854 correspond to samples with higher transcript levels. For Northern blots in parts B and D, microRNA161 (miR161) and a heterochromatic-region 24 nt siRNA (siRNA02) are shown as loading controls.

In the plant body, retrotransposons such as *Athila6* are tightly epigenetically suppressed by heritable symmetric DNA methylation and RdDM [3]. In each case of 21 nt siRNA854 accumulation (*met1*, *ddm1* and pollen) loss of TE epigenetic silencing is known to occur [11]–[12], [34]–[35]. To determine if the *Athila6* retrotransposon is specifically activated in these mutants and pollen, we performed real-time quantitative RT-PCR (qRT-PCR) and found that in *met1* and *ddm1* mutants and wt pollen, *Athila6* transcript accumulation is significantly increased compared to wt Col whole seedlings, leaf and inflorescence tissue (Figure 1A). We used qRT-PCR to measure *Athila6* expression (Figure 1), as well as a separate *Athila6* primer set specific to the microRNA stem-loop structure previously proposed (Figure S1) [29]. Both primer sets provided similar data, showing that neither the proposed stem-loop nor flanking *Athila6* region transcripts accumulate in wt Col seedlings, leaves or inflorescences, while both regions are expressed in *ddm1*and *met1*mutants. In addition, *Athila6* transcripts accumulate in wt Col pollen (Figure 1A). Compared relatively, pollen has considerably less *Athila6* transcript accumulation than either *ddm1* or *met1* mutants, perhaps due to the fact that pollen TE reactivation only occurs in the pollen vegetative nucleus, one of three nuclei expressing mRNA in mature pollen [12].

To examine siRNA854 accumulation in more detail, we performed small RNA Northern blots and found in wt Col and *ddm1* and *met1* inflorescences, a 24 nt version containing the siRNA854 sequence accumulates, while 21–22 nt versions of this sequence accumulate only in *ddm1* and *met1* (Figure 1B), as well as in pollen (Table 1). We then probed this Northern blot with a 300 bp siRNA854-flanking region of *Athila6* (*Athila6* 3′ probe) and found that this region also produces other 24 and 21–22 nt siRNAs at levels comparable to those of siRNA854. These results demonstrate that the production of 21–22 nt siRNAs from this entire region is under the same epigenetic regulation as siRNA854, and combined with the results of deep sequencing of small RNAs from *ddm1*, *met1* and pollen [12]–[13], demonstrates that siRNA854 is one member of a larger region of siRNA production. Our data refutes previous data that characterized a specific microRNA produced from this region of the *Athila6* retrotransposon [29].

The phenotype of *ddm1* mutant plants becomes more severe in progressive generations. Second generation homozygotes for the recessive *ddm1-2* allele (*ddm1* F2) display little to no morphological phenotype, while after propagation as a homozygote for four additional generations (*ddm1* F6), leaf size and infertility phenotypes are severe [36]. Figure 1C shows that increasing transcript accumulation of the *Athila6* retrotransposon is associated with the progression of *ddm1* from the F2 to F6 generation. To determine if the different transcript levels of *Athila6* directly correlate with the accumulation of 21–22 nt siRNA854 and flanking 21–22 nt *Athila6* 3′ siRNAs, we examined siRNA854 accumulation by Northern blot in *ddm1* F2 and F6 individuals. F6 *ddm1* individuals produce increased levels of siRNA854 and other *Athila6* 3′ siRNAs compared to F2 generation *ddm1* individuals (Figure 1D). These data, together with the transcript accumulation and siRNA accumulation in *met1* and pollen (Figure 1A and 1B, Table 1), suggests that the epigenetic activation and level of *Athila6* steady-state transcripts directly and positively correlates with the accumulation level of *Athila6* 21–22 nt siRNAs, including siRNA854.

### siRNA854 biogenesis is atypical for a retrotransposon siRNA and requires *RDR6*, *DCL2*, *DCL4*, and *AGO1*

**Figure 2:**
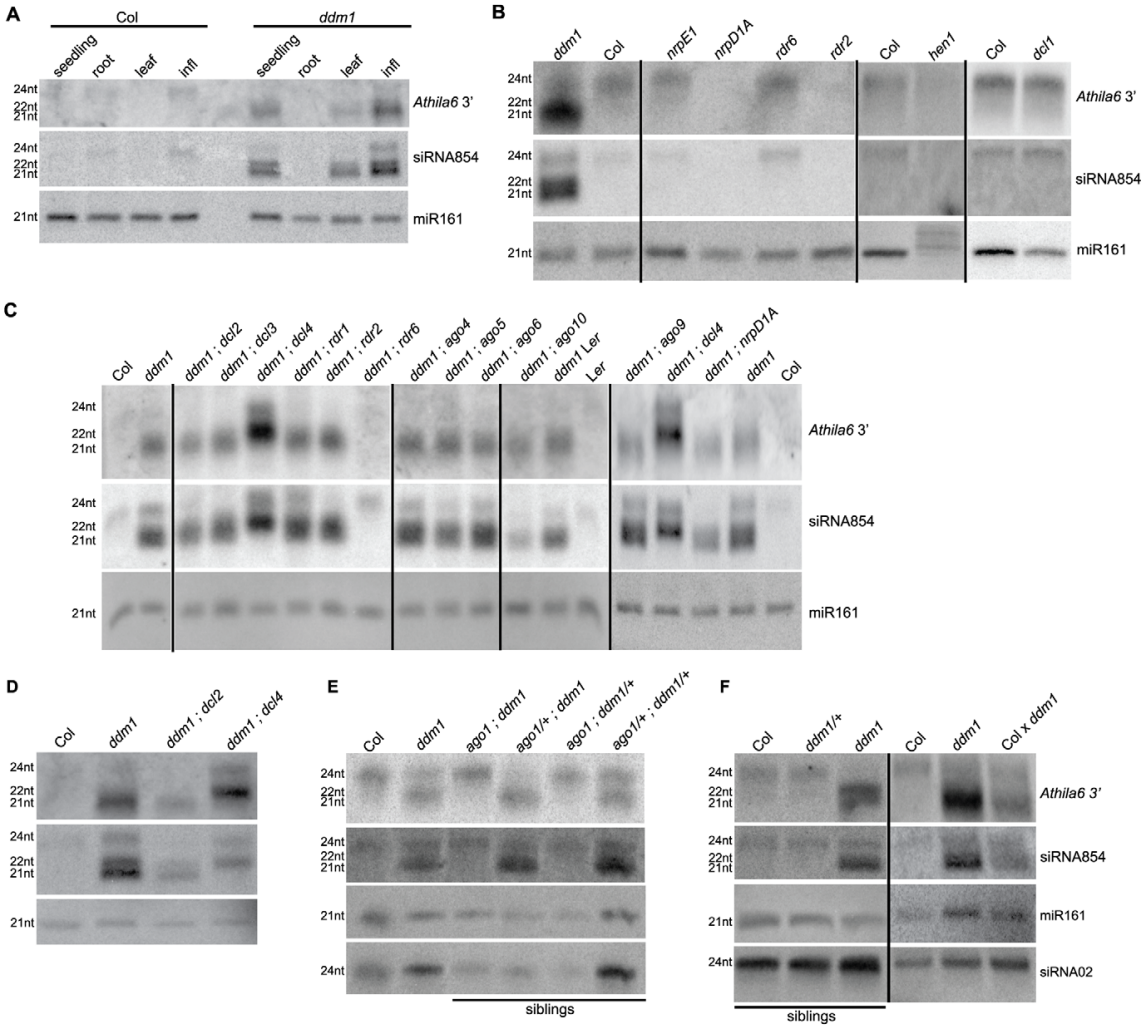
Biogenesis of *Athila6* siRNAs and siRNA854. All parts correspond to Northern blots detecting siRNA854 and a flanking region of *Athila6* 3′ of the *gag/pol* protein coding region. (A) In wt Col, only the 24 nt versions of siRNA854 and *Athila6* siRNAs accumulate, while in *ddm1* seedling, leaf and infl, 21 nt and 22 nt versions of siRNA854 and flanking *Athila6* siRNAs accumulate. (B) Biogenesis pathway of the 24 nt *Athila6* siRNAs. *hen1*, *rdr2* and *nrpD1A* mutants fail to accumulate *Athila6* 24 nt siRNAs. (C) Biogenesis pathway of the 21–22 nt *Athila6* siRNAs that accumulate upon transcriptional activation. *ddm1* double mutants were generated for mutants involved in known siRNA production pathways. 21–22 nt *Athila6* 3′ siRNAs and siRNA854 fail to accumulate in *ddm1;rdr6* double mutants, while the 21 nt siRNAs shift to 22 nt in *ddm1;dcl4* double mutants. (D) Higher resolution Northern showing that the 21 nt version of siRNA854 and *Athila6* 3′ siRNAs are absent in *ddm1;dcl4* double mutants, while the 22 nt version is absent in *ddm1;dcl2* double mutants. (E) The accumulation of *Athila6* 3′ siRNAs in a *ddm1;ago1* segregating family produced from *ddm1* homozygote P1 plants. *AGO1* is necessary for the accumulation of 21–22 nt siRNA854 and *Athila6* 3′ siRNAs from both *ddm1* homozygotes and *ddm1* heterozygotes (*ddm1*/+). (F) 21–22 nt *Athila6* 3′ siRNAs and siRNA854 accumulate in *ddm1* heterozygotes produced by crossing wt to an individual homozygous for the recessive *ddm1-2* allele (Col x *ddm1*). *ddm1/+* heterozygous plants produced from parents that have not been homozygous for *ddm1* for at least 6 generations do not accumulate *Athila6* 21–22 nt siRNAs and are shown as a control (*ddm1/+* in segregating family).

To determine the biogenesis mechanism responsible for producing the 21–22 nt versions of *Athila* siRNAs and siRNA854, we first screened four tissues of wt Col and *ddm1* mutant plants and determined that *Athila6* 21–22 nt siRNAs are not detected in wt Col seedlings, roots, leaves or inflorescences (Figure 2A). *Athila6* 21–22 nt siRNAs are most easily detectable in *ddm1* inflorescence tissue, while leaf and seedling tissues have lower relative levels, and the siRNAs are undetectable in roots (Figure 2A). In wt Col, 24 nt *Athila6* siRNAs weakly accumulate in the root and inflorescence (Figure 2A), and these inflorescence 24 nt siRNAs are dependent on the *PolIV* component *NRPD1A*, *RDR2*, and the small RNA-modifying protein HEN1 (Figure 2B). *HEN1* is responsible for the accumulation of both microRNAs and siRNAs [37], while the requirement of *NRPD1A* and *RDR2* demonstrates that, like other known 24 nt TE siRNAs, *Athila6* 24 nt siRNAs are generated by the RdDM pathway which is responsible for maintaining epigenetically silenced regions of the genome [21], [38]. The 24 nt siRNA854 accumulation in wt Col inflorescences is not dependent on *RDR6*, the *PolV* component *NRDE1*, or the microRNA processing *DCL1* (Figure 2B). Contrary to previously published results, these data demonstrate that there is no siRNA854 version in wt Col inflorescences that is dependent on the microRNA machinery.

To determine the small RNA biogenesis pathway responsible for producing 21–22 nt siRNA854 and *Athila6* siRNAs when *Athila* is epigenetically activated, we generated 12 double mutant combinations with *ddm1*, using mutants for genes with known roles in the various *Arabidopsis* small RNA biogenesis pathways, such as different *AGO*, *DCL* and *RDR* genes. Double mutant inflorescences were used to assay the accumulation of siRNA854 (Figure 2C). As some of these double mutants are in Col x Landsberg *erecta* (L*er*) mixed genetic backgrounds, as a control we confirmed that L*er ddm1* mutants also accumulate *Athila6* siRNAs, while wt L*er* does not. We found that the RdDM pathway responsible for producing *Athila6* 24 nt siRNAs involving *NRPD1A* and *RDR2* does not generate the 21 nt or 22 nt siRNA854. This represents a change in siRNA biogenesis pathways for *Athila* siRNAs, as their epigenetic reactivation results in a new set of proteins responsible for the 21–22 nt siRNA production. We determined that *RDR6* function is required for both 21 nt and 22 nt siRNA854 accumulation, as in *ddm1;rdr6* double mutants neither of these siRNAs accumulate (Figure 2C), while *RDR6* is not responsible for the 24 nt version of these siRNAs (Figure 2B). *RDR6*'s involvement suggests that an *Athila6* transcript is copied into dsRNA, which is required for 21–22 nt siRNA production. We also found that in *ddm1;dcl4* double mutants, the 21 nt siRNA854 is absent, while increased levels of the 22 nt and 24 nt versions are detected. There are well-described hierarchical relationships among *DCL2*, *DCL3*, and *DCL4*. When *DCL4* is not present to generate 21 nt siRNAs, *DCL2* primarily substitutes for this function and generates 22 nt siRNAs, while *DCL3* substitutes for *DCL4* to a lesser degree and produces 24 nt siRNAs [39]. Conversely, *ddm1;dcl2* double mutants lose the 22 nt version of *Athila6* siRNAs, including siRNA854 (Figure 2D), demonstrating that the 21 nt and 22 nt versions of siRNA854 that accumulate in *ddm1* mutants are generated by the activity of *DCL4* and *DCL2*, respectively. The production of 21 nt or 22 nt siRNA854 in either *ddm1;dcl2* or *ddm1;dcl4* double mutants suggests that the processing by DCL4 and DCL2 proteins occurs after RDR6 converts the *Athila6* transcript into dsRNA. In addition, the proteins responsible for the biogenesis of the 24 nt version of siRNA854 and, separately, for the 21–22 nt version are identical to those responsible for generating the corresponding sizes of the flanking *Athila6* siRNAs, further indicating that siRNA854 is not solely or specifically cleaved from this region.

To determine which *Argonaute* protein(s) are responsible for siRNA854 accumulation, we tested *ddm1* double mutants with *ago1*, *ago4*, *ago5*, *ago6* and *ago10*. *ddm1* double mutants with *ago4*, *ago5*, and *ago6* did not result in loss of siRNA854, and *ago10* double mutants displayed only reduced accumulation (Figure 2C). In the *ddm1;ago1* double mutant both the 21 nt and 22 nt versions of siRNA854 fail to accumulate (Figure 2E), demonstrating that *AGO1* is essential for 21–22 nt siRNA854 accumulation. The requirement of *RDR6*, *DCL2*, *DCL4* and *AGO1* suggests that either the known VIGS pathway of post-transcriptional degradation of viral RNAs, or the tasiRNA pathway is responsible for *Athila6* 21–22 nt siRNA biogenesis.

While generating the *ddm1* double mutant plants, we encountered an unusual pattern of inheritance of *Athila6* siRNAs, which stems from the atypical genetic inheritance of *ddm1* mutants. For example, both the *ddm1* mutant phenotype and *Athila6* expression become more severe over increasing generations (Figure 1C) [36]. In addition, *ddm1*/+ heterozygote plants produced by crossing a plant homozygous for the *ddm1-2* recessive allele to wt Col inherit epigenetically decondensed and transcriptionally uncontrolled chromatin from the *ddm1* parent, which is not fully restored in the *ddm1*/+ heterozygote [40]–[42]. This mutant chromatin in a *ddm1*/+ heterozygous individual continues to express TEs [42]. In Figure 2F we demonstrate that a *ddm1*/+ heterozygote produced from a *ddm1* homozygous parent (Col x *ddm1* in Figure 2F) still produces 21–22 nt siRNA854 and *Athila6* 3′ siRNAs, although to a considerably lower level than the *ddm1* homozygote. This is in contrast to a *ddm1*/+ heterozygote that was not the progeny of a homozygous parent, but has been backcrossed to wt Col for six generations while being maintained as a heterozygote. In this *ddm1*/+ heterozygote (*ddm1*/+ in segregating family, Figure 2F) the amount of mutant chromatin inherited from the *ddm1* homozygous parent has been diluted away by segregation in each generation of crossing to wt Col, demonstrating that there is an effect of the parent's genotype on the production of *Athila6* siRNAs in *ddm1*/+ heterozygous plants. The requirement of *AGO1* for the production of 21–22 nt siRNA854 in *ddm1*/+ heterozygotes was demonstrated using an F2 family segregating for *ago1* and *ddm1*, which was produced from a *ddm1* homozygous P1 individual (Figure 2E). In *ago1* mutants that are *ddm1*/+ heterozygotes (*ago1;ddm1/+*), neither 21 nor 22 nt versions of siRNA854 accumulate, while they do in the corresponding *ago1/+;ddm1/+* double heterozygote siblings. These data demonstrate that *AGO1* is necessary for siRNA854 accumulation in *ddm1* mutants and in the progeny of *ddm1* homozygotes.

### siRNA854 accumulation represses reporter gene transcripts with the *UBP1b* 3′UTR

**Figure 3:**
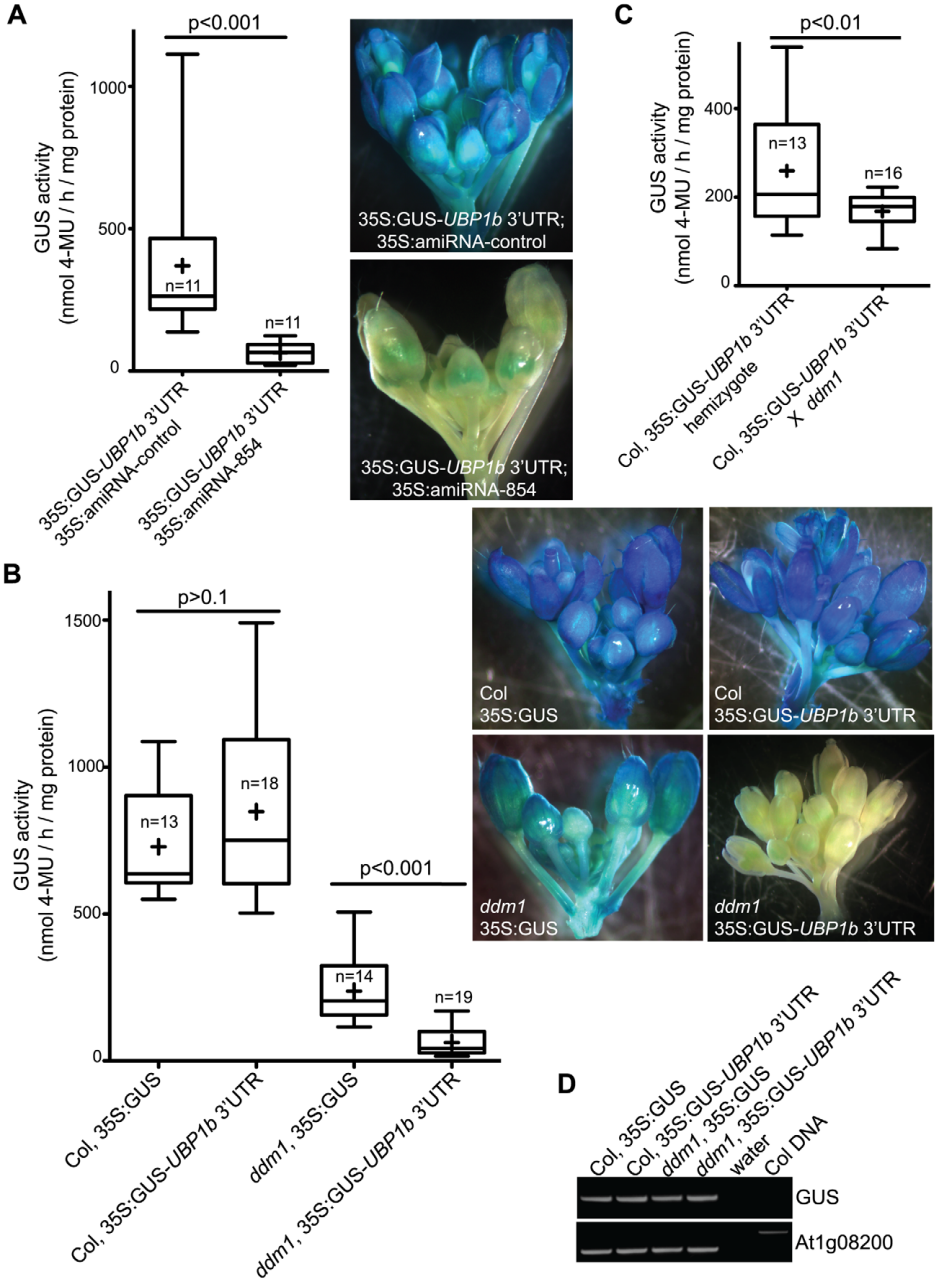
Accumulation of 21–22 nt siRNA854 negatively regulates transgene transcripts with the *UBP1b* 3′ UTR. (A) Plants homozygous for a transgene constitutively expressing the GUS reporter protein fused to the *UBP1b* 3′ UTR (35S:GUS-*UBP1b* 3′UTR) were transformed with an artificial microRNA (amiRNA) with the siRNA854 sequence (35S:amiRNA-854), or a second control sequence that does not target *UBP1b* (35S-amiRNA-control). GUS activity was monitored using a quantitative assay (left) and inflorescence staining (right). Plants expressing the siRNA854 sequence as an artificial microRNA show significantly reduced GUS levels. (B) Col and *ddm1* plants homozygous for a constitutively expressed GUS transgene (35S:GUS) or the same reporter transgene with the *UBP1b* 3′ UTR from part A. As in A, GUS activity was monitored using a quantitative assay (left) and plant staining (right). Wt Col plants show no differential regulation between the two transgene variations, while in the *ddm1* mutant background the GUS activity of the *UBP1b* 3′ UTR transgene is significantly less than the control 35S:GUS transgene. (C) A 35S:GUS-*UBP1b* 3′UTR transgene in the wt Col background was crossed to a *ddm1* homozygote, and the GUS activity was measured in the F1 plant. GUS activity of the same hemizygous transgene in the wt Col background is shown as a control. (D) RT-PCR of the same transgenic individuals from part B. The GUS activity differences observed in part B are not reflected in transgene transcript levels, demonstrating that this regulation is not due to post-transcriptional mRNA degradation. In A, B and C, the box plot whiskers represent the minimum and maximum of the dataset, the top and bottom of the box are the 75^th^ and 25^th^ percentile (respectively), the middle line is the median, and + is the mean. The number of individuals assayed (n) is shown in or near the box.

The 21 nt version of siRNA854 was previously predicted to target the 3′UTR of the *UBP1b* gene in four locations using modified criteria that allows for non-canonical or ‘wobble’ G:U base pairing [29]. G:U base pairing has been demonstrated to be tolerated in microRNA target sites, even within the critical first 7 base pairs (bp) or ‘seed’ pairing region [43]. However, the targeting of the 3′UTR by small RNAs was not previously shown directly, and complementarity of siRNA854 to the *UBP1b* 3′UTR relies heavily on non-canonical base pairing and lacks a strong 7 bp seed-pairing region (shown in Figure S2). To directly test if the 21 nt siRNA854 sequence has the ability to target the *UBP1b* 3′UTR, we took advantage of the fact that wt Col inflorescences do not accumulate 21–22 nt siRNA854 (Table 1, Figure 1, Figure 2). To directly examine the role of the siRNA854 sequence, we constructed plants constitutively expressing a GUS reporter gene with the GUS mRNA fused to the *UBP1b* 3′UTR and transformed these plants with artificial microRNA (amiRNA) constructs expressing the siRNA854 sequence, or an unrelated sequence as a control, from the *Arabidopsis* microRNA319a stem-loop transcript [44]. Quantitative assays to detect GUS activity, as well as qualitative plant staining, demonstrate that the plants with the control amiRNA have high levels of GUS activity, while plants expressing the siRNA854 sequence from a microRNA stem-loop display significantly lower levels of GUS activity (Figure 3A). These data demonstrate that although the alignment of siRNA854 to the *UBP1b* 3′UTR lacks a strong seed pairing region and relies on G:U base pairing, the 21 and/or 22 nt siRNA854 sequence can target the *UBP1b* 3′UTR resulting in decreased reporter protein accumulation.

The developmental expression profiles of *UBP1b* and the six *Athila6* elements on the Affymetrix ATH1 gene expression microarray are negatively correlated, with *UPB1b* expressed highly in all wt tissues except mature pollen, specifically where *Athila6* activation occurs (Figure S3A). To determine if the increased levels of endogenous 21–22 nt siRNA854 observed when *Athila6* is epigenetically activated can regulate the *UBP1b* 3′UTR, we transformed both wt Col and *ddm1* plants with either the GUS-*UBP1b* 3′UTR transgene from Figure 3A, or a control transgene without the *UBP1b* 3′UTR. We assayed GUS activity in plants homozygous for the transgenes and found that in wt Col, the presence of the *UBP1b* 3′UTR did not affect GUS activity (Figure 3B). In contrast, when this same analysis was previously published, the same GUS-*UPB1b* 3′UTR transgene in a wt Col plant resulted in little to no GUS protein production in leaves and inflorescences [29]. Our data, which demonstrate no inhibition of the *UBP1b* 3′UTR in wt Col leaves and inflorescences, are supported by the fact that there is no 21/22 nt siRNA854 detected in leaves or inflorescence by either Northern blot (Figure 1, Figure 2A) or small RNA deep sequencing (1 read in a combined 6.6 million)(Table 1).

In *ddm1* mutants, the GUS-*UBP1b* 3′UTR and GUS control (no 3′UTR) transgenes both display reduced GUS activity (Figure 3B). It remains enigmatic why the constitutively expressed GUS transgene without a 3′UTR has reduced expression in *ddm1* compared to wt Col. However, the presence of the *UBP1b* 3′UTR resulted in a significant reduction of GUS activity compared to the no 3′UTR control in *ddm1* (Figure 3B). To make sure that position effects of these transgene insertions were not the cause of this differential regulation, we crossed a wt Col plant with the *UBP1b* 3′UTR transgene that displayed high GUS activity to a *ddm1* homozygote, as the resulting heterozygote will have siRNA854 accumulation (Figure 2F). The GUS activity in this *ddm1* heterozygote is significantly reduced compared to both the wt Col homozygous GUS transgene parent and to wt Col plants heterozygous for the same GUS transgene (Figure 3C). Therefore, the regulation of the *UBP1b* 3′UTR is sensitive to the levels of siRNA854, with either the production of this sequence as an amiRNA, or accumulation of this sequence as an siRNA in *ddm1* resulting in repression of GUS activity. We determined that all of the transgenes in either wt Col or *ddm1* from Figure 3B have GUS transcripts that accumulate to similar levels (Figure 3D), indicating that the regulation of these transgene transcripts is not due to post-transcriptional degradation of the GUS RNA, but is likely rather due to the inhibition of translation of these mRNAs.

**Figure 4:**
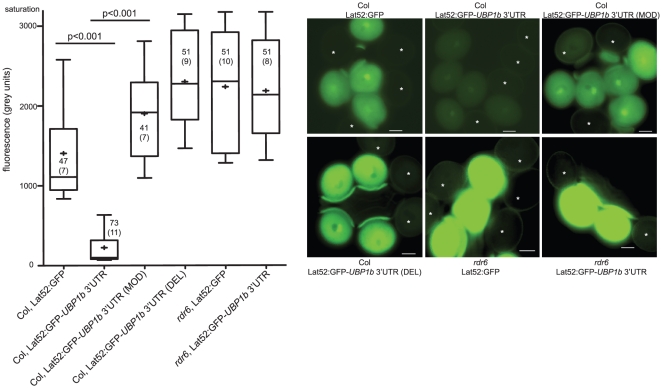
Accumulation of 21–22 nt siRNA854 in wt pollen regulates transgene transcripts with the *UBP1b* 3′UTR. GFP transgenes with pollen-specific expression were used to assay the activity of endogenous siRNA854 in pollen. Background corrected GFP fluorescence levels are shown on the left, while representative pollen images are on the right. On each image, an asterisk marks non-fluorescent pollen grains that did not inherit the transgene from the hemizygous parent that were used for background correction. The addition of the *UBP1b* 3′UTR to a GFP transgene results in reduced fluorescence. Abrogation of the perfectly complementary base pairing in the predicted siRNA854 target sites of the *UBP1b* 3′UTR (MOD), or removal of these target sites altogether (DEL), alleviates this repression. The repression of the *UBP1b* 3′ UTR in pollen is lost in *rdr6* mutant plants. Box plot whiskers represent the 90^th^ and 10^th^ percentile of the dataset, the top and bottom of the box are the 75^th^ and 25^th^ percentile (respectively), the middle line is the median, and + is the mean. Number of pollen grains measured and the number of transgenic individuals they came from (in parentheses) is shown in or near the box. Scale bars = 10 µm.

In addition to the increased levels of siRNA854 in *ddm1* mutants, siRNA854 also accumulates in wt Col pollen (Table 1). To determine if endogenous siRNA854 in pollen is able to regulate the *UBP1b* 3′UTR, we performed similar transgene reporter assays as above in wt Col pollen. We used a pollen vegetative cell promoter to drive GFP and added one of three different 3′UTRs to these reporter transgenes. GFP fluorescence was quantitatively measured by subtracting the fluorescence of segregating pollen grains that did not inherit the transgene from the fluorescence of pollen grains that did inherit the transgene. With no 3′UTR, transgene protein accumulates, and a moderate level of fluorescence is observed (Figure 4). When the wt *UBP1b* 3′UTR is added to this transgene, significantly less fluorescence is observed, likely due to the accumulation of siRNA854 in wt Col pollen. To test if the binding of siRNA854 was specifically responsible for this regulation, we generated a version of the *UBP1b* 3′UTR that lacks all four of the siRNA854 predicted target sites, resulting in a shorter 3′UTR (shown in Figure S2). This deleted 3′UTR transgene (DEL transgene) resulted in significantly increased fluorescence compared to the wt *UBP1b* 3′UTR (Figure 4). We also produced a *UBP1b* 3′UTR variation of the same length as the wt *UBP1b* 3′UTR, in which each of the perfectly complementary base pairs in all four of the siRNA854 predicted target sites have been switched to bases that do not show complementarity (or G:U pairing) with siRNA854 (Figure S2). Pollen grains with the base-modified 3′UTR (MOD transgene) on the GFP transcript display significantly increased fluorescence compared to the wt *UBP1b* 3′UTR (Figure 4), demonstrating that these bases are necessary for the regulation of the *UBP1b* 3′UTR. Pollen from both the MOD and DEL 3′UTR transgenes display fluorescence levels even higher than the control lacking a 3′UTR, likely due to the ability of the *UBP1b* 3′UTR, when not targeted by small RNAs, to stabilize transcripts or promote their translation. Lastly, we transformed the GFP transgene with and without the wt *UBP1b* 3′UTR into *rdr6* mutants. We observed that the expression of Lat52:GFP (no 3′UTR) in *rdr6* mutant pollen is higher than that of the same transgene in wt Col pollen (Figure 4). This difference is likely due to the role of *RDR6* in post-transcriptional silencing of transgenes [45]. We speculate that the wt Col Lat52:GFP transgene is subject to a certain low amount of post-transcriptional regulation mediated by *RDR6*. When this transgene is present in *rdr6* mutant plants, this post-transcriptional regulation does not occur, resulting in higher expression of the transgene compared to wt Col. Interestingly, we did not observe a reduction in pollen fluorescence for the Lat52:GFP-*UBP1b* 3′UTR transgene in *rdr6* compared to the no-3′UTR control transgene in *rdr6* (Figure 4), demonstrating that *RDR6* is necessary for the targeting of the *UBP1b* 3′UTR in pollen. The combined data from Figure 3 and Figure 4 demonstrate that the *RDR6*-dependent accumulation of siRNA854 and base pairing with the *UBP1b* 3′UTR target sites are required for the inhibitory regulation of *UBP1b* 3′UTR reporter genes.

### siRNA854 represses endogenous *UBP1b* in pollen

We aimed to determine if siRNA854 has a regulatory effect on the endogenous *UBP1b* gene or transcript. To aid our characterization of *UBP1b* we isolated two mutant *upb1b* alleles, which are in the Ws background. *UBP1b* insertion alleles result in a lack of polyadenylated transcripts, although un-spliced and non-polyadenylated transcripts are still produced (Figure S4).

**Figure 5:**
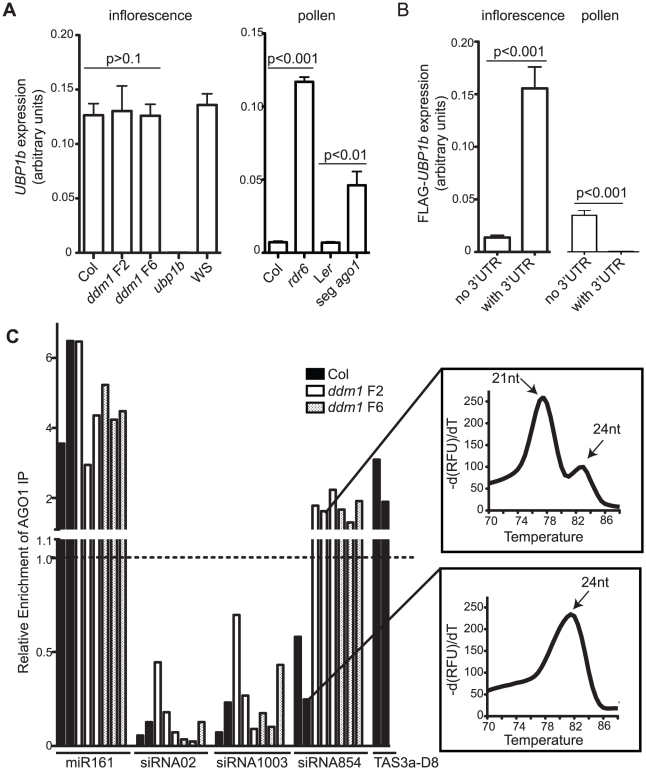
Tissue-specific regulation of *UBP1b* by siRNA854. (A) qRT-PCR of *UBP1b* in inflorescence tissue and mature pollen. In inflorescence tissue, *UBP1b* transcript accumulation is not affected in *ddm1* mutants. In wt Col pollen, *UBP1b* expression significantly increases in *rdr6* mutants and in a pool of pollen that is segregating 1∶1 *ago1* mutant pollen (seg *ago1*). (B) qRT-PCR of FLAG-tagged *UBP1b* transgenes with and without the *UBP1b* 3′UTR and under control of their native promoters. In inflorescence tissue, the addition of the *UBP1b* 3′UTR results in increased transcript levels. In pollen, the *UBP1b* promoter is active, and addition of the *UBP1b* 3′UTR results in significantly decreased levels of mRNA. (C) qRT-PCR of small RNAs from AGO1-IP biological replicates demonstrating that in the plant body of *ddm1* mutants siRNA854 is enriched in AGO1 protein complexes, while it is not in wt Col. Relative enrichment values over 1.0 indicate AGO1-enrichment, whereas relative enrichment values under 1.0 indicate no enrichment. Relative enrichment was calculated based on amplification of the input sample for each IP. MiR161 and TAS3a-D8 are shown as positive controls while siRNA02 and siRNA1003 are 24 nt siRNA negative controls not bound by AGO1. qRT-PCR melting curves for siRNA854 amplification products from the AGO1-IP demonstrate that the non-enriched siRNA854 in wt Col is the larger 24 nt version (higher melting temperature), while the AGO1-enriched siRNA854 in *ddm1* is the 21–22 nt version (lower melting temperature).

First, we wondered if the sequence similarity between the 21, 22 or 24 nt versions of siRNA854 was directing DNA methylation to the *UBP1b* 3′UTR through the RdDM pathway, as a possible mechanism of epiallele production. We have determined that the DNA methylation status of the *UBP1b* 3′UTR is not altered in *ddm1* inflorescences relative to wt Col (Figure S5A). Next, we utilized microarray data and RT-PCR to analyze *UPB1b* transcript levels, and we found they accumulate to the highest levels in inflorescence tissue, intermediate levels in seedling and leaf tissues, and either to extremely low levels or not at all in wt Col pollen (Figure S3). We measured *UBP1b* transcript accumulation in *ddm1* mutants at two developmental time points: inflorescence buds and mature pollen. In inflorescence tissue, the transcript accumulation of *UPB1b* is not significantly altered in *ddm1* F2 or *ddm1* F6 inflorescences (Figure 5A). Therefore, qRT-PCR expression analysis and DNA methylation analysis both demonstrate that in inflorescence tissue there is no transcriptional or post-transcriptional effect of siRNA854 on *UBP1b* transcript accumulation. We continued to assay *UBP1b* in inflorescence tissue of wt Col and *ddm1* supposing that the regulation may be on the translational level, as was observed for the inflorescences of the GUS-*UBP1b* 3′UTR transgene transcript in Figure 3. We assayed two known microRNA-induced alterations to transcripts associated with translational regulation (reviewed in [46]). We determined that in *ddm1* inflorescence tissue the polyA tail length of *UBP1b* is unaffected, and uncapped transcript does not accumulate (Figure S5B and S5C). Without the availability of a specific antibody to assay endogenous UBP1b protein accumulation, we can provide no direct evidence that endogenous *UPB1b* transcripts are regulated by the elevated siRNA854 levels that accumulate in *ddm1* inflorescences.

In contrast to inflorescence tissue, the transcript accumulation of *UBP1b* in pollen is regulated at the post-transcriptional level by siRNA854. In wt Col pollen, the *UBP1b* transcript does not accumulate (Figure S3). To determine if this is a consequence of the increased levels of siRNA854 in pollen, or if the *UBP1b* promoter is simply not active in mature pollen, we performed qRT-PCR in *rdr6* mutant plants as well as from plants heterozygous for *ago1*. Plants homozygous for the recessive *ago1-11* allele do not produce pollen, so we used an *ago1-11*/+ heterozygote that produces pollen segregating 1∶1 wt and mutant for *ago1*. In both *rdr6* pollen and *ago1* segregating pollen there is a significant increase in *UBP1b* transcript accumulation, with *rdr6* having a >16-fold increase (Figure 5A). This demonstrates that the *UBP1b* promoter is active in pollen, but the transcripts are subject to post-transcriptional degradation. Attempts at identifying siRNA854-induced cleavage sites in the *UPB1b* 3′UTR from inflorescence and pollen were inconclusive (data not shown), likely due to the high rate of non-small RNA-induced processing and degradation of the endogenous *UBP1b* 3′UTR detected in whole genome degradome analysis [47]–[48].

To determine if the *UBP1b* 3′UTR is specifically responsible for the differential *UBP1b* accumulation in inflorescence and pollen, we generated two transgenes with the native *UBP1b* promoter and coding region, with and without the 3′UTR. This transgene also has a 5′ FLAG epitope tag to distinguish it from the endogenous *UBP1b*. We found that the presence of the *UBP1b* 3′UTR in inflorescence tissue increases the transcript accumulation levels >11-fold, presumably by stabilizing this transcript (Figure 5B). In wt Col pollen the *UBP1b* promoter is active, and without the 3′UTR this transcript accumulates to levels 4-fold less compared to inflorescence tissue. However, in contrast to inflorescence tissue, the addition of the *UBP1b* 3′UTR resulted in >73-fold reduced transcript accumulation in wt pollen. Together, these data demonstrate that in wt Col pollen the presence of the *UBP1b* 3′UTR causes a decrease in *UBP1b* transcript accumulation.

The mature pollen grain contains two sperm cells with highly condensed chromatin imbedded into the larger vegetative cell, which displays a chromatin-decondensed vegetative nucleus. Communication between the vegetative cell and imbedded sperm cells has been previously hypothesized to occur (reviewed in [49]). To determine in which cell post-transcriptional silencing in the pollen grain is taking place, we aimed to decipher where in the mature pollen grain the repression of the *UBP1b* 3′UTR is occurring. Since we demonstrated that both *RDR6* and *AGO1* are necessary for the repression of the endogenous *UBP1b* transcript in the mature pollen grain (Figure 5A), we examined the localization of the RDR6 and AGO1 proteins by fusing them to GFP and expressing them from their native promoters. We found that both of these proteins localize to the nucleus and cytoplasm of the pollen vegetative cell and are not detectable in sperm cells (Figure S6), in agreement with mined microarray data from purified sperm cells [50]. The pollen vegetative cell is also the location of epigenetic TE reactivation [12], suggesting that the activation of *Athila6*, cleavage into siRNAs and potentially the repression of the *UBP1b* 3′UTR are all occurring in this cell.

Since a functional AGO1 protein is required for the accumulation of siRNA854 (Figure 2E), and *UBP1b* transcript levels increase in segregating mutant *ago1* pollen (Figure 5A), we aimed to determine if siRNA854 is incorporated into AGO1 protein complexes. We performed an immunoprecipitation (IP) of AGO1 complexes using a commercially available AGO1 antibody and purified the incorporated small RNA. To verify the specificity of the AGO1 antibody, we demonstrated that this antibody does not detect any other proteins by first performing a Western blot on protein extracts from Col, *Ler*, and *ago1-11* inflorescences. We found that the AGO1 antibody yields no cross-reactive bands (Figure S7A). Additionally, we used Western blot analysis to confirm the success of the IP by both detecting the presence of AGO1 protein in the IP Input extract and AGO1 IP samples, and observing the absence of AGO1 protein in the no antibody IP control (Figure S7B). After the IP, we purified the AGO1-bound small RNAs and used qRT-PCR to assay levels of siRNA854, a known AGO1-incorporated microRNA (miR161), a known AGO1-incorporated tasiRNA (TAS3a-D8), and two 24 nt heterochromatic siRNAs not present in AGO1 complexes (siRNA02 and siRNA1003) [51]–[52]. We found no AGO1-IP enrichment of siRNA854 or the control siRNAs, siRNA02 and siRNA1003, in the wt Col plant body, while we did detect enrichment of the control microRNA miR161 and control tasiRNA TAS3a-D8 (Figure 5C). In contrast, in *ddm1* F2 and F6 plants we found enrichment of siRNA854 in AGO1. Analysis of the melting curves generated from the products of the qRT-PCR demonstrate that the background levels of siRNA854 from wt Col plants are the larger 24 nt version (which have higher melting temperatures) compared to the AGO1-enriched 21–22 nt siRNA854 from *ddm1* plants (lower melting temperatures)(Figure 5C). The level of enrichment of siRNA854 in AGO1 complexes in *ddm1* mutants is not as high as miR161, but is more similar to the level of enrichment of the tasiRNA TAS3a-D8 in wt Col (Figure 5C), likely due the fact that both siRNA854 and TAS3a-D8 are single siRNAs from transcripts that generate multiple siRNAs through the activity of RDR6 and DCL4. In addition, we detected no difference in enrichment levels between *ddm1* F2 and *ddm1* F6 plants. However, since there are higher levels of siRNA854 in the *ddm1* F6 plants (Figure 1D) and input (non-IP) sample that was used for normalization, there are likely more AGO1 protein complexes interacting with siRNA854 in F6 *ddm1* plants compared to the F2 generation. These data demonstrate that only when epigenetically activated, the *Athila6*-generated 21–22 nt siRNA854 is incorporated into AGO1, and this complex is responsible for the regulation of the *UBP1b* transcript.

### Altered stress-regulation in *ddm1* mutants

**Figure 6:**
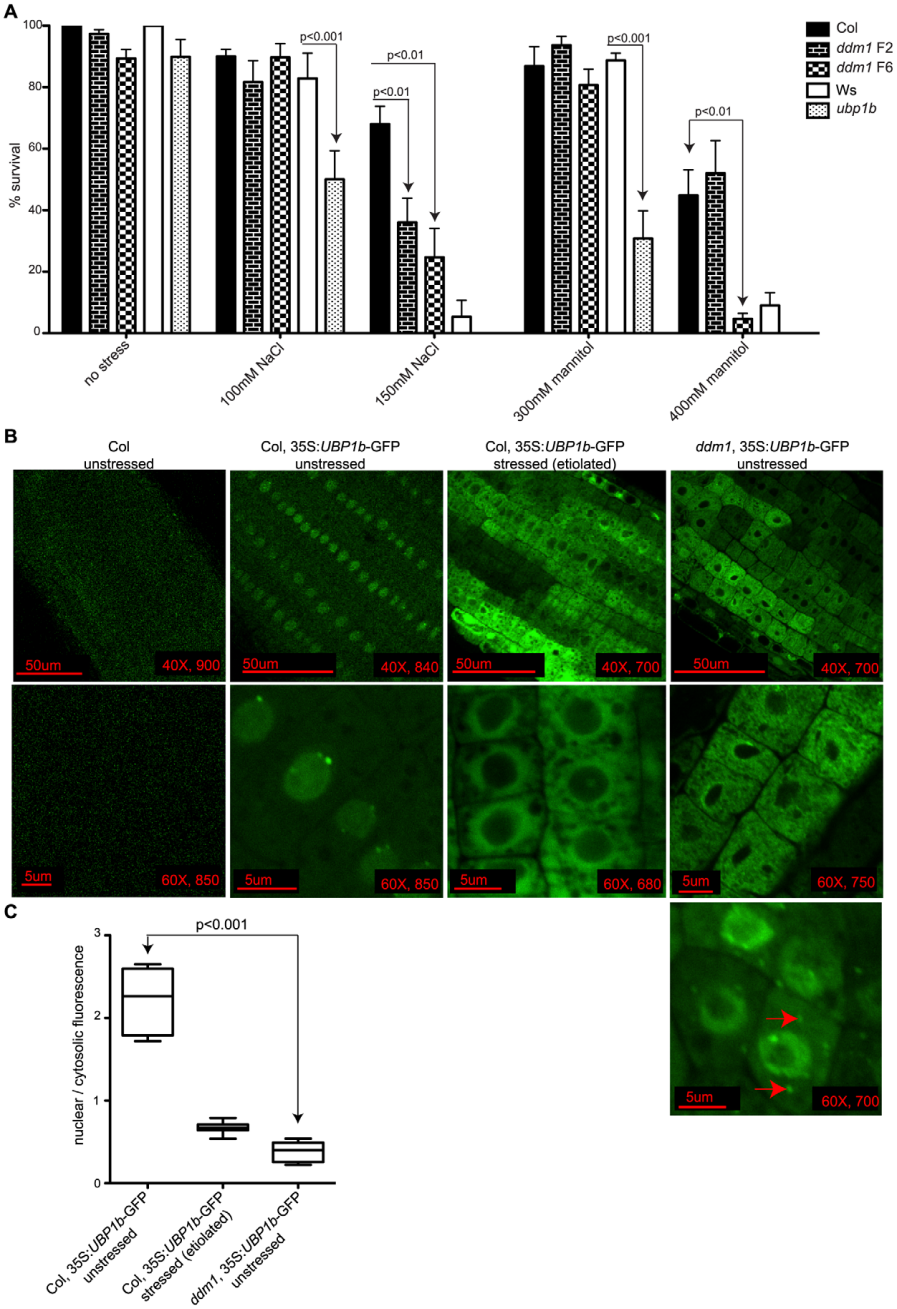
UBP1b protein localization and stress sensitivity of *ddm1*. (A) Survival of plants grown under conditions of ionic and osmotic stress. Seeds were spotted on plates containing no stressor, increasing levels of NaCl or increasing levels of mannitol. After 11 days the number of surviving plants were counted. Wt plants of the Ws background are more sensitive to the stressors tested than wt Col. Compared to the corresponding wt Ws control, *ubp1b* mutants are more sensitive to both ionic (100 mM NaCl) and osmotic (300 mM mannitol) stresses. No *upb1b* seedlings survived under stress conditions of 150 mM NaCl or 400 mM mannitol. *ddm1* F6 generation plants are also sensitive to both ionic (150 mM NaCl) and osmotic (400 mM mannitol) stresses compared to wt Col. (B) Imaging of a constitutively expressed UBP1b protein fused to GFP (without its native 3′ UTR) in the root cell elongation zone. In unstressed wt Col seedling roots the UBP1b-GFP protein is localized to the nucleus, where specific peri-nuclear bright foci are observed. Growth of the same plants under stress conditions (due to etiolation) results in the relocalization of the UBP1b-GFP fusion protein from the nucleus into the cytosol. In unstressed *ddm1* mutants, the UBP1b-GFP protein accumulates in the cytosol, while occasional cells display distinct cytoplasmic foci (arrows). For each image, scale bars, magnification, and exposure time (in ms) are shown. (C) Measurements of fluorescence intensity were taken from the lines in B, and a ratio of nuclear to cytosolic fluorescence was calculated. The localization of UBP1b-GFP fluorescence is significantly different in unstressed wt Col compared to unstressed *ddm1*.

TIA-1 has a known role in the sensing and response to cellular stress, and mutant cells unable to form stress granules display increased sensitivity to stress [53]–[55]. We have experimentally determined that the germination and growth of *ubp1b* mutant plants are also sensitive to both ionic (+NaCl) and osmotic (+mannitol) stress conditions compared to its corresponding wt background Ws, particularly when grown on 100 mM NaCl or 300 mM mannitol (Figure 6A). In addition, wt Ws itself is more sensitive to these stresses than wt Col, as at higher NaCl or mannitol concentrations, wt Col survives but wt Ws does not. Since *ddm1* plants have increased levels of siRNA854 (Table 1, Figure 1), and siRNA854 can target the *UBP1b* 3′UTR (Figure 3, Figure 4), we tested *ddm1* seedlings to determine if they display a similar stress sensitivity as *upb1b* mutant plants. Plants that have been homozygous *ddm1* for six generations (*ddm1* F6) are significantly more sensitive than the corresponding wt Col for both ionic and osmotic stress, while *ddm1* F2 is only sensitive to ionic stress (Figure 6A). Similar to *ubp1b* mutants, *ddm1* mutant plants are sensitive to ionic and osmotic stress conditions. These data, combined with our demonstrated regulation of *UBP1b* levels by siRNA854 (Figure 5), suggest that the *ddm1* stress sensitivity acts directly through epigenetic activation of *Athila6* and production of siRNA854, which results in the post-transcriptional and/or translational repression of *UBP1b*.

The mammalian homolog of *UBP1b* is TIA-1, an RNA binding protein localized to the nucleus that moves into the cytoplasm and aggregates into stress granules upon induction of stresses such as treatment with arsenite, glucose deprivation, and viral infection [56]–[58]. To visualize the sub-cellular localization of the UBP1b protein in response to cellular stress in *Arabidopsis*, as well as to determine the influence of *ddm1* on this process, we expressed an siRNA854-resistant version of *UBP1b* (without its native 3′UTR) fused to GFP, under constitutive expression. In wt Col seedling roots this protein is localized to the nucleus, with distinct bright peri-nuclear foci observed (Figure 6B). However, when transformed into *ddm1* plants, this same transgene displayed cytosolic fluorescence (Figure 6B). We aimed to induce stress in both the wt Col and *ddm1* UBP1b-GFP lines; however, the ionic and osmotic conditions from Figure 6A inhibited growth in the *ddm1* plants. Therefore, we experimentally determined that germination and growth in the dark (etiolation) would generate a UPB1b protein stress response, without killing the plant. We germinated and grew the wt Col UBP1b-GFP plants under etiolation conditions and observed a shift in the sub-cellular localization of UBP1b-GFP, as fluorescence accumulated around the periphery of the nucleus and in the cytoplasm (Figure 6B). This change in sub-cellular distribution of UBP1b-GFP under a condition of stress (etiolation) is analogous to the movement of TIA-1 out of the nucleus under stress conditions [59]. In etiolated *ddm1* plants, the UBP1b-GFP fluorescence pattern is the same as in non-stressed *ddm1* plants (data not shown). We quantified these fluorescence patterns by measuring the amount of nuclear vs. cytoplasmic fluorescence and found a statistically significant difference between the localization of UBP1b-GFP in unstressed wt Col compared to unstressed *ddm1* (Figure 6C). The unstressed *ddm1* fluorescence pattern resembles the stressed wt Col roots (Figure 6B and 6C). Additionally, in a very small number of *ddm1* cells (roughly 1/1000), we observe a second accumulation pattern that displays distinct cytoplasmic foci reminiscent of mammalian stress granules (red arrows, Figure 6B), as well as fluorescence at the periphery of the nucleus. Together, these data suggest that the translocation of the UBP1b protein in *ddm1* mutant cells is a response to an intracellular stress as a result of the *ddm1* mutation, perhaps due to altered gene expression in *ddm1* mutants, or due to the loss of DNA methylation, loss of repressive histone modifications, and activation of TEs [4], [60].

## Discussion

### The epigenetic control of *Athila6* and siRNA854

SiRNA854 is a gene-regulating endogenous siRNA that is produced from the *Athila6* family of LTR retrotransposons, and its accumulation is strictly dependent upon the transcriptional epigenetic activation of the *Athila6* element. *Athila* elements, as well as nearly all other types of TEs in wt Col *Arabidopsis*, are normally transcriptionally silenced and are associated with DNA methylation and 24 nt siRNAs involved in maintaining the transcriptionally silenced state [7], [13]. The 21–22 nt versions of siRNA854 are only produced upon *Athila6* transcriptional activation, such as in *ddm1* and *met1* mutants, or in the vegetative cell of wt pollen. Like *Athila6* activity itself, siRNA854 production displays two unusual epigenetic *trans*-generational inheritance patterns. First, siRNA854 is produced in a *ddm1*/+ heterozygote that was generated from a *ddm1* homozygote, and the pathway of this accumulation remains dependent on *AGO1* in the *ddm1*/+ heterozygote. Second, there is a positive correlation between the increasing levels of *Athila6* mRNA and accumulation of *Athila6* 21–22 nt siRNAs (including siRNA854) as *ddm1* mutants are propagated from the F2 to the F6 generation. The progressively increasing transcript levels in *ddm1* F2 to F6 generations could be due to increased rates of transcription, perhaps due to progressive loss of heterochromatin control, such as *Athila* DNA methylation, each generation. The positive correlation in *Athila* mRNA and siRNA levels suggests that the *Athila6* transcript is the limiting factor in siRNA854 production, and that at least some *Athila6* mRNA transcripts can accumulate although the 3′ region of *Athila6* is degraded into siRNAs and amplified using a RNA-dependent RNA polymerase. This correlation could potentially be due to two different subsets of elements that increase in expression from the *ddm1* F2 to F6 generation, one subset that is cleaved into siRNAs and one subset that is not. Alternatively, *Athila6* transcripts may be converted into siRNAs at a very slow rate, allowing time for the transcripts to accumulate before degradation.

### SiRNA854 biogenesis

Most *Arabidopsis* TEs lose siRNA production when epigenetically activated. There is an unknown factor that causes some TEs, such as *Athila*, to produce siRNAs even when transcriptionally active. In contrast to some TE families such as *ATGP1*, which simply retains 24 nt siRNAs when activated, *Athila* is one of only very few element families identified to date that produces 21–22 nt siRNAs when epigenetically activated and expressed [12], [15]–[16]. The production of 21–22 nt siRNAs represents a shift in small RNA biogenesis pathways from *PolIV*-dependent 24 nt siRNAs processed by the RdDM pathway, to a post-transcriptional silencing pathway that presumably uses *PolII*-derived transcripts and involves *DCL2*, *DCL4*, *RDR6* and *AGO1*, with at least siRNA854 eventually incorporated into AGO1. These *DCL*, *RDR* and *AGO* proteins act in both the tasiRNA and VIGS pathways, and *Athila* processing shows hallmarks of both. For example, the VIGS pathway likely acts on *Athila6* transcripts, as *Athila* has evolved from an LTR retrovirus, and, due to the conservation of the *envelope* protein coding domain, the *Athila4* subfamily has even been classified as an *Arabidopsis* endogenous retrovirus [61]. *Athila6* siRNAs may be produced via the VIGS pathway; however, siRNA854's ability to regulate *UBP1b* in *trans* is functionally more similar to the tasiRNA pathway. Therefore, we defer classifying *Athila6* 21–22 nt siRNAs as either tasiRNAs or VIGS siRNAs.

The classification of *Athila6* 21–22 nt siRNAs as either tasiRNAs or VIGS siRNAs perhaps can be resolved once the initiation of their production is understood. We currently have three models for how the initiation of *Athila6* 21–22 nt siRNAs may occur. First, the secondary structure of the *Athila6* transcript, particularly in the region of siRNA production, may fold back into hairpin-like structures, producing a substrate for DCL4 cleavage. Second, overlapping sense and antisense *Athila6* transcripts may result in the formation of a dsRNA trigger, as *Athila* elements accumulate in nested arrays of elements near the centromere that favor the production of read-through transcripts (reviewed in [62]). A pathway of natural antisense transcript siRNA (nat-siRNA) production exists in *Arabidopsis*; however, *PolIV* and *RDR2* are required for this pathway [63]–[64], and we have experimentally determined that these proteins are not required for *Athila6* 21–22 nt siRNA accumulation. A third proposed mechanism of 21–22 nt *Athila6* siRNA initiation may be similar to tasiRNA initiation and the initiation of islands of 21–22 nt siRNA accumulation in maize and rice. MicroRNA(s) may initiate the cleavage of an *Athila6* transcript, causing the production of *RDR6*- and *DCL4*-dependent siRNAs. In rice, the production of 21 nt phased siRNAs is dependent first on microRNA cleavage, and then on *OsDCL4* for production, and these siRNAs preferentially accumulate in male reproductive organs [65]. One microRNA that shows potential seed region complementarity to *Athila6* is the 22 nt microRNA845b; however, *Athila* siRNAs are produced on either side of the predicted *Athila6* cleavage site, and our experiments to date provide no evidence that microRNA845b is required for *Athila6* 21–22 nt siRNA biogenesis (data not shown). In addition to acting downstream of siRNA854 production, it is currently unknown if AGO1 acts upstream of DCL4 and RDR6 to initiate *Athila6* transcript cleavage, but it is likely that a 22 nt siRNA initiates the RDR6-dependent amplification of *Athila6* siRNAs [66]. If initiated by a tasiRNA-like mechanism, tasiRNA-like phasing should be detected in the *Athila6* siRNA production. We have not detected any such phasing of *Athila6* siRNAs (data not shown), but this analysis is complicated by the 12 nearly identical *Athila6* elements that carry siRNA854, and dozens more *Athila* elements that are cleaved into siRNAs at the same time. If each element that produces 21–22 nt siRNAs is correctly phased, but not in the same register as each other, our analysis would detect no phasing. Therefore, although we have identified AGO1, RDR6 and DCL4 as necessary for the accumulation of siRNA854, the trigger for *Athila6* siRNA initiation remains to be elucidated.

### The regulation of *UBP1b* by siRNA854

We used the *UBP1b* 3′UTR in reporter assays to demonstrate that whenever we observe the accumulation of the 21–22 nt siRNA854 sequence (in *ddm1* mutants, wt pollen, or expressed as an amiRNA), we observe decreased reporter protein accumulation. Expression of the siRNA854 sequence as an amiRNA in the vegetative tissue of wt plants demonstrates that the potentially complicating secondary effects occurring from loss of heterochromatin control in *ddm1* mutant plants and wt pollen are not responsible for repression of the *UPB1b* 3′UTR. Both the siRNA854-amiRNA and endogenous siRNA854 are able to inhibit protein production from reporter transcripts bearing the *UBP1b* 3′UTR, and in pollen this regulation is dependent on the siRNA854 target sites in the 3′UTR, as well as on *RDR6*.

We have also demonstrated that the endogenous *UBP1b* transcript is regulated by siRNA854. In inflorescence tissue, this regulation is likely on the translational level, and this result is supported by the translational regulation of the GUS-*UBP1b* 3′UTR transcript in inflorescences. In contrast, in mature pollen we detect post-transcriptional regulation of the endogenous *UBP1b* transcript by siRNA854. This pollen post-transcriptional regulation of the endogenous *UPB1b* transcript is under the control of *RDR6* and *AGO1*, suggesting that the accumulation of siRNA854 is necessary for this regulation. The basis of the switch from translational control in inflorescence tissue to post-transcriptional control in pollen remains puzzling. One possibility is that the four predicted target sites for siRNA854 in the *UBP1b* 3′UTR are not equally available to base pair in inflorescence tissue compared to pollen. Therefore, in pollen the interaction of siRNA854 with one target site may cause transcript cleavage, while in inflorescence tissue the interaction with a different target site may lead to translational inhibition. Lastly, the observation of 21–22 nt siRNA854 still present in *ddm1* heterozygotes produced from *ddm1* homozygous parents suggests that there may be a *trans*-generational epigenetic component to the regulation of *UBP1b*, as *UBP1b* may continue to respond to *Athila* activity even when the plant is no longer homozygous for *ddm1*. This *trans*-generational regulation was observed with the GUS-*UBP1b* 3′UTR transgene in an individual heterozygous for the recessive *ddm1-2* allele, due to the inheritance of mutant chromatin from the parental plant.

### *Athila* and stress

Under the stress condition of etiolation, the UBP1b-GFP protein traffics from the nucleus and accumulates in the cytoplasm. In unstressed *ddm1* mutants, this siRNA854-resistant form of UBP1b is also located in the cytoplasm, suggesting that some aspect of the *ddm1* mutation triggers this stress-sensing change in protein location, independent of siRNA854. It is currently unknown which characteristic(s) of the *ddm1* mutation triggers this response, as *ddm1* mutants display aberrant control of genic epialleles, global TE activation, TE mobilization, and general chromatin decondensation [4], [35], [60], [67]. Conversely, *ddm1* mutant seedlings show a phenotype similarly sensitive to ionic and osmotic stress as *upb1b* mutants. Several studies have shown that TEs are reactivated during stress conditions [68]–[69]; however, in this case TEs are regulating the stress-responsive pathway. Taken together, these data suggest that an antagonism exists between the *UBP1b*-induced stress response, which is activated in *ddm1* mutants, and *Athila6*, which inhibits this response by targeting *UBP1b* through siRNA854. This antagonism may also exist in animal cells, as some DNA viruses generate microRNAs that specifically target the *UBP1b* homologue TIA-1 mRNA [70], while other RNA viruses specifically target stress granule proteins for proteolysis [71], presumably for the same reason that *Athila* targets *UBP1b*. Since TIA-1 is known to repress the activity of some animal viruses and retrotransposons through the formation of stress granules [72]–[73], we speculate that the same is true for *Athila*. Therefore, we envision a three-layered host repression of *Athila* activity. First, transcriptional regulation dependent on DNA methylation epigenetically silences *Athila*. Second, when transcriptionally active, *Athila* mRNA accumulation is inhibited by the post-transcriptional regulation mediated by the tasiRNA/VIGS siRNA pathway components *DCL2*, *DCL4*, *RDR6* and *AGO1*. Third, we speculate that *Athila* transcripts may be translationally inhibited due to their sequestration in stress granules, targeted by the UBP1b protein. Transcripts in stress granules are not degraded, but are not translated due to their separation from active ribosome complexes (reviewed in [54]). Akin to a virus encoding a suppressor of gene silencing [20], *Athila* may encode siRNA854 to inhibit UBP1b protein formation and interfere with the function of this translational-level repression.

### TE regulation of genes

AGO1 is known to mediate gene regulation via siRNAs in the tasiRNA pathway [74]–[75]. We have demonstrated that an siRNA which is not part of one of the four known tasiRNA producing loci (*TAS1*-*4*), but rather part of an epigenetically regulated TE, is able to act on genic transcripts in *trans* in a similar fashion to a tasiRNA. We think the key aspect of this regulation is the incorporation of siRNA854 into AGO1. AGO1 is the main *Argonaute* protein responsible for gene regulation in *Arabidopsis* (reviewed in [76]). This protein is likely unable to distinguish between an siRNA generated from a transcriptionally reactivated TE and one generated from a tasiRNA precursor transcript, at least in the case of siRNA854. Sequencing from AGO1 immunoprecipitations has demonstrated a higher than expected level of siRNAs [52], [77], providing evidence that AGO1 is likely regulating both genic transcripts using microRNAs, as well as viral, TE or other repeat transcripts via siRNAs and post-transcriptional silencing. Further investigation is required to understand if and how AGO1 protein complexes determine which siRNAs should target genic mRNAs in *trans* and which should not. Therefore, the possibility currently exists that siRNA854 does not act alone, and the genome-wide regulation of many transcripts is altered by TE or viral siRNAs loaded into AGO1. It is an intriguing possibility, since both TE epigenetic activation and viral infection lead to a series of still unexplained changes in gene regulation and phenotype. In fact, one longstanding enigmatic viral symptom of the Cucumber mosaic virus was recently found to be caused by a viral satellite siRNA targeting a host gene in *trans* [20]. In animals, many viruses encode microRNAs that target cellular genes to generate a favorable cellular environment [78]. In order to gain this same advantage, plant TEs may carry sequences that do not require a microRNA stem-loop structure, but utilize a different mechanism by co-opting the tasiRNA/VIGS siRNA biogenesis machinery to regulate a diverse set of cellular transcripts.

## Materials and Methods

### Plant material

The mutant alleles used in this study are listed in Table S1. All mutants are in the Col background except *ago1* (L*er*), *ago10* (L*er*), *hen1-1* (L*er*), *ubp1b* (FLAG_298B04)(Ws), *upb1b* (FLAG_071F09)(Ws), and *ddm1* L*er*. Plants were grown under standard long-day growth chamber conditions. Etiolated and stress-test plants were stratified for 3 days at 4°C and grown for 11 days on 1/2X MS media+Gamborg's vitamins with supplemented sucrose in 16 hours of light per day, with the exception of etiolated seedlings, which were grown without light. For the stress-test analysis, the number of plants surviving after 11 days was counted. Fifty seedlings of each genotype for each condition were grown, and the analysis was replicated three or more times.

### qRT–PCR

Total RNA was extracted using TRIzol reagent (Invitrogen) or the RNeasy Plant Kit (Qiagen). Total RNA was DNAseI treated and reverse transcribed using an oligo-dT primer and SuperScript III Reverse Transcriptase (Invitrogen). qRT-PCR was performed with iQ SYBR Green Supermix (BioRad Laboratories) using 3 technical replicates each of 3 or more biological replicates. qRT-PCR primers are shown in Table S1. qPCR reactions were annealed at 60°C unless otherwise noted. Since most standard qRT-PCR control genes are not highly expressed in pollen, the relative expression values for all experiments were calculated based on the expression of the experimentally validated control gene At1g08200. qPCR was performed on a CFX96 thermocycler and the results analyzed on the CFX Manager Software package (BioRad Laboratories). Relative expression was calculated using the ‘delta-delta method’ formula 2^−[ΔCP sample−ΔCP control]^, where 2 represents perfect PCR efficiency. Statistical significance was calculated using unpaired T-tests.

### Small RNA Northern blots

Total RNA was extracted using TRIzol reagent (Invitrogen), and small RNA was enriched by polyethylene glycol precipitation. The quantity of small RNA loaded in each lane ranged from 16–60 µg between blots, though the same amount of RNA was loaded per lane on each blot for comparison between samples. We accounted for the equal loading and sizes of small RNAs by re-probing our Northern blots with a known 21 nt microRNA (miR161) and/or a known 24 nt siRNA (siRNA02). In addition, our small RNA Northern blot analysis is supported by independent small RNA deep sequencing data [33]. Gel electrophoresis, blotting and cross-linking were performed as in Pall *et al.* [79]. Probes for siRNA854, miR161, and siRNA02 were generated by 5′ labeling DNA oligonucleotides with P^32^-ATP, whereas the probe for *Athila* 3′ was generated by randomly degrading a P^32^-UTP labeled *in vitro* transcribed RNA as in [80]. Sequences of DNA oligonucleotides and primers for generating the *in vitro* transcription template are listed in Table S1.

### Transgene construction and analysis

The 35S:amiRNA-siRNA854 transgene was generated by cloning the sequence 5′GATGAGGATAGGGAGGAGGAG into the microRNA319a stem-loop transcript as in [44]. This transcript was sub-cloned into the 35S promoter binary plasmid pB2GW7. The wt version of the *UBP1b* 3′UTR was amplified from the wt Col genome, and the 35S:GUS-*UBP1b* 3′UTR transgene was produced as in [29]. GUS staining was performed as in [81]. For GUS protein activity quantification, protein was quantified using the *DC* assay (BioRad Laboratories), and 1 mg was used to assay the cleavage of MUG into fluorescent 4-MU as in [82]–[83]. Fluorescence was measured in 96-well format with a Tecan-SpectraFluor Plus microplate reader, and the specific activity of GUS in each sample was calculated as nmol of 4-MU formed per hour per mg of protein (nmol 4-MU/h/mg). RT-PCR of these lines was performed with oligo-dT primed cDNA for 28 cycles of PCR using primers listed in Table S1.

The modified and deleted versions of the *UBP1b* 3′UTR were synthesized by IDT. The Lat52 promoter-driven GFP-*UPB1b* 3′UTR transgene was constructed by cloning the either the wt *UBP1b* 3′UTR, Modified *UBP1b* 3′UTR or Deleted 3′UTR version into the SacI site at the end of the mGFP coding sequence of the binary plasmid pMDC107, and then by adding the Lat52 promoter to the KpnI site upstream of mGFP in these clones. GFP fluorescence quantification was performed on a Nikon C2 confocal microscope with the NIS-Elements software suite (Nikon Corporation). GFP quantification was performed with the same microscope settings (exposure time, laser power) on the same day. Subtraction of the fluorescence of pollen grains that did not inherit the GFP transgene from the same hemizygous plant negated the background pollen auto-fluorescence.

The FLAG-*UBP1b* transgene was constructed by adding the FLAG epitope sequence to the 5′ end of the *UBP1b* CDS as in [51]. This FLAG-*UBP1b* fragment was amplified and cloned into pENTR/D-TOPO (Invitrogen). The *UBP1b* promoter and 5′UTR were inserted 5′ to the FLAG tag, and the *UBP1b* 3′UTR was inserted 3′ of the *UBP1b* coding region by In-Fusion Recombination (Clontech). Subsequent constructs were recombined into pBGW by Gateway LR Recombination (Invitrogen). Specific qRT-PCR primer sets detecting the FLAG-*UBP1b* transgene are shown in Table S1. Attempts at identifying the FLAG-UBP1b protein using a FLAG-epitope antibody were repeatedly unsuccessful.

The 35S:*UBP1b*-GFP transgene was generated by cloning the *UBP1b* coding region into the binary plasmid pK7FWG2. Seedlings were grown on 1/2X MS media for 11 days before their roots were analyzed by confocal microscopy. The ratio of nuclear to cytosolic fluorescence was calculated by using the NIS-Elements software (Nikon Corporation) by manually defining the average fluorescence touching an analysis line transecting the nucleus and cytosol of an individual cell. The ratios of 25 cells were examined per condition.

All *Arabidopsis* transformations were performed using Agrobacterium strain GV3101 and standard laboratory techniques. Statistical significance was calculated using unpaired T-tests.

### AGO1 immunoprecipitation and small RNA analysis

The AGO1 protein was immunoprecipitated as follows using a commercially available polyclonal AGO1 antibody (Agrisera AB) specific to the unique N-terminal peptide of AGO1, which has been demonstrated to lack cross-reactivity with various over-expressed AGO proteins [84]. Inflorescence tissue was ground with liquid nitrogen and homogenized in 2 ml extraction buffer (100 mM Tris-HCl (pH 7.5), 150 mM NaCl, 1 mM EDTA, and 5 mM DTT) containing 1 tablet/10 mL protease inhibitor cocktail (Roche) per gram of tissue. In a standard immunoprecipitation reaction, *Arabidopsis* protein extract was pre-cleared by incubation with 10 µl of goat anti-rabbit magnetic beads (NEB). Pre-cleared extracts were then incubated overnight with goat anti-rabbit magnetic beads pre-incubated with 1 µg α-AGO1. All washes were performed with extraction buffer. Immunoprecipitated, mock-immunoprecipitated and input sample RNA was isolated using TRIzol (Invitrogen). 125 ng of each RNA sample was subjected to polyA tailing, cDNA synthesis, and qRT-PCR according to the QuantiMir product specifications (System Biosciences Incorporated). The PCR was annealed at 61.5°C and performed on 2–3 biological replicate immunoprecipitations for each genotype tested, each one having 3 technical qPCR replicates. Each qRT-PCR IP C(t) value was normalized to the amplification of its own input sample, using the ‘delta-delta method’ formula 2^−[ΔCP IP−ΔCP Input]^, where 2 represents perfect PCR efficiency.

## Supporting Information

Figure S1 Transcript accumulation of the proposed microRNA854 stem loop structure. qRT-PCR using primers specific for the previously proposed microRNA854 stem loop structure [29]. Transcripts from this region of the *Athila6* retrotransposon do not accumulate in vegetative tissues of wt Col. Similar to other regions of *Athila6*, transcript accumulation is activated in *ddm1* and *met1* mutants.
(EPS)

Figure S2 Base pairing of siRNA854 to the wt, MOD and DEL versions of the *UBP1b* 3′ UTR. The *UBP1b* 3′ UTR is shown from 5′ to 3′. The four sites of the *UBP1b* 3′UTR targeted by siRNA854 predicted by Arteaga-Vázquez *et al.* are shown [27]. In the MODified 3′ UTR variant, all perfect complementary base pairing was replaced. In the DELeted 3′ UTR variant, the entire target sites have been removed, resulting in a shorter 3′ UTR.
(EPS)

Figure S3 Expression analysis of *UBP1b* in wt Col. (A) Mined data from microarray experiments performed and normalized by Schmid *et al.* were accessed through AtGenExpress (http://jsp.weigelworld.org/expviz/expviz.jsp) [79]. GC-RMA normalized values for the accumulation of *UBP1b* and the six *Athila6A* elements on the ATH1 gene expression microarray are shown. (B) Expression level of the *UBP1b* mRNA in seedlings, rosette leaves, inflorescence and mature pollen as in part A are shown on a linear scale. (C) RT-PCR of *UBP1b* mRNA accumulation in wt Col tissues. Polyadenylated transcripts do not accumulate in pollen, while they do accumulate in inflorescences, leaves and seedlings. RT-PCR was performed on 200 ng of total RNA, reverse transcribed with an oligo-dT primer or random hexamers using Superscript III Reverse Transcriptase (Invitrogen). PCR was performed for 28 cycles.
(EPS)

Figure S4 Analysis of *ubp1b* mutant plants. RT-PCR of *ubp1b* homozygous mutant plants. For both the FLAG298B04 and FLAG071F09 insertion alleles, *UBP1b* is still transcribed, but not spliced correctly, and the transcript is not polyadenylated. These insertions are in the Ws background. RT-PCR was performed on 200 ng of total RNA reverse transcribed with an oligo-dT primer or random hexamers using Superscript III Reverse Transcriptase (Invitrogen). PCR was performed for 28 cycles.
(TIF)

Figure S5 Transcript-level regulation of *UBP1b* by siRNA854 is not observed in inflorescence tissue. (A) Bisulfite PCR analysis of the DNA methylation levels of the *UBP1b* 3′UTR in wt Col and *ddm1*. DNA was treated by the manufacturer's instructions using the EpiTech Bisulfite Kit (Qiagen), amplified using the primers in Table S1, TOPO-TA cloned into pCR4 (Invitrogen) and sequenced. Both the sense DNA strand (top) and antisense DNA strand (bottom) were interrogated separately. Each circle represents a cytosine in the DNA sequence, with the color of the circle corresponding to the sequence context of the cytosine (CG = red, CHG = blue, CXX = green, H = A,T,C). Closed circles represent methylated cytosines, while open circles represent unmethylated. The location of the cytosine corresponds to the map of the *UBP1b* last exon and 3′ UTR shown below for each DNA strand. The locations of the four predicted siRNA854 target sites on the *UBP1b* 3′ UTR are shown as red lines on the maps. (B) 3′ RACE-PAT analysis of the polyA tail length of the *UBP1b* transcript shows no differences between wt Col and *ddm1*. Transcripts from the *ubp1b* mutant are not polyadenylated. This technique was performed as in [80] using primers shown in Table S1. (C) Modified 5′RACE-PCR detecting full-length uncapped transcripts shows no difference in the level of *UBP1b* full-length uncapped transcripts between wt Col and *ddm1*. Uncapped *UBP1b* transcripts accumulate in the *ubp1b* mutant. A modified GeneRacer (Invitrogen) 5′RACE protocol was performed using 5 µg TRIzol-isolated total RNA. RNA was ligated to a 5′ RNA adaptor by T4 RNA Ligase I and reverse transcribed with an oligo-dT primer and SuperScript III Reverse Transcriptase (Invitrogen). Uncapped transcripts were detected by two rounds of nested PCR using adaptor-specific and gene-specific primers, listed in Table S1.
(EPS)

Figure S6 Pollen localization of *RDR6* and *AGO1*. (A) Expression values from microarray data mined from Borges *et al.* of purified sperm cells and whole mature pollen [45]. *UBP1b*, *RDR6* and *AGO1* transcripts are not enriched in sperm cells. *AGO5* is shown as a control of a known sperm-specific protein [81]. (B) Fluorescence microscopy images of mature pollen grains expressing GFP fused to the RDR6, AGO1 or AGO5 protein, each under control of their own native promoters. The transgenes were generated by cloning the promoters and open reading frames of the proteins (including introns) into the binary plasmid pMDC107. Transgenes were transformed into a line expressing RFP in the pollen vegetative nucleus (VN-RFP) [82]. Plants hemizygous for the transgene were used for analysis, and pollen grains that did not inherit the transgene are marked with an asterisk. pAGO5:AGO5-GFP is shown as a control for a protein that has known sperm cell localization [81]. In the images of pAGO1:AGO1-GFP and pRDR6:RDR6-GFP, dark shadows of the sperm cells in the vegetative cell cytoplasm can be seen. Scale bars are 20 microns. (C) Complementation of the *rdr6* mutant narrow leaf phenotype with the RDR6p:RDR6-GFP transgene from part B. All plants are 14 days old. The pAGO1:AGO1-GFP transgene did not complement the *ago1-11* seedling phenotype (data not shown).
(TIF)

Figure S7 The AGO1 antibody is specific to the AGO1 protein. (A) Western blot of Col, L*er*, and *ago1-11* inflorescence tissue protein extract using the same AGO1 antibody as in Figure 5. The *ago1-11* allele contains a single nucleotide polymorphism in a splice acceptor site that causes exon skipping and results in a weak allele that retains some AGO1 function [85]. Proteins sized 50–200 kDa were transferred to nylon, which was stained with Ponceau-S to show even loading and then blotted with α-AGO1. No cross-reactive bands were detected. The *ago1-11* allele has decreased protein levels, but a small amount of AGO1 protein is still produced. (B) Western blot of Col inflorescence protein extract (Input), no antibody immunoprecipitation control (Mock IP) and AGO1 IP using the same AGO1 antibody as in Figure 5. The Input sample contains 1/15 of the total input protein compared to the Mock IP and AGO1 IP samples. The Western blot only detects AGO1 protein in Input and AGO1 IP samples, while no AGO1 protein is detected in the Mock IP.
(EPS)

Table S1 PCR primers and mutant alleles used in this report.
(XLS)
