# Supplementary figures and images for: Gene Expression and Stress Response Mediated by the Epigenetic Regulation of a Transposable Element Small RNA
Source: PLoS Genet. 2012 Feb 9;8(2):e1002474. doi: 10.1371/journal.pgen.1002474 (PMC3276544; doi:10.1371/journal.pgen.1002474)

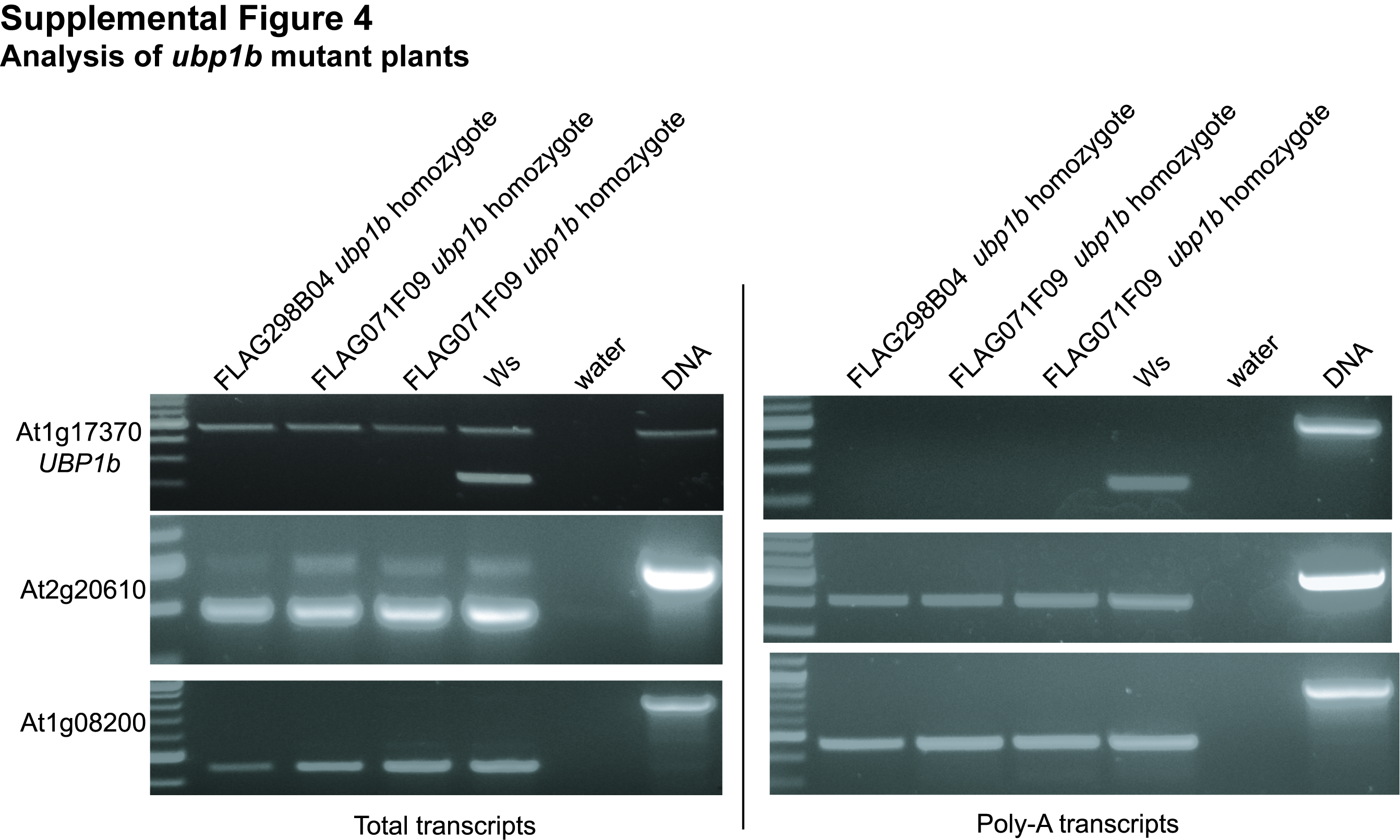

Supplement: Figure S4 — Analysis of ubp1b mutant plants. RT-PCR of ubp1b homozygous mutant plants. For both the FLAG298B04 and FLAG071F09 insertion alleles, UBP1b is still transcribed, but not spliced correctly, and the transcript is not polyadenylated. These insertions are in the Ws background. RT-PCR was performed on 200 ng of total RNA reverse transcribed with an oligo-dT primer or random hexamers using Superscript III Reverse Transcriptase (Invitrogen). PCR was performed for 28 cycles. (TIF) [file pgen.1002474.s004.tif]

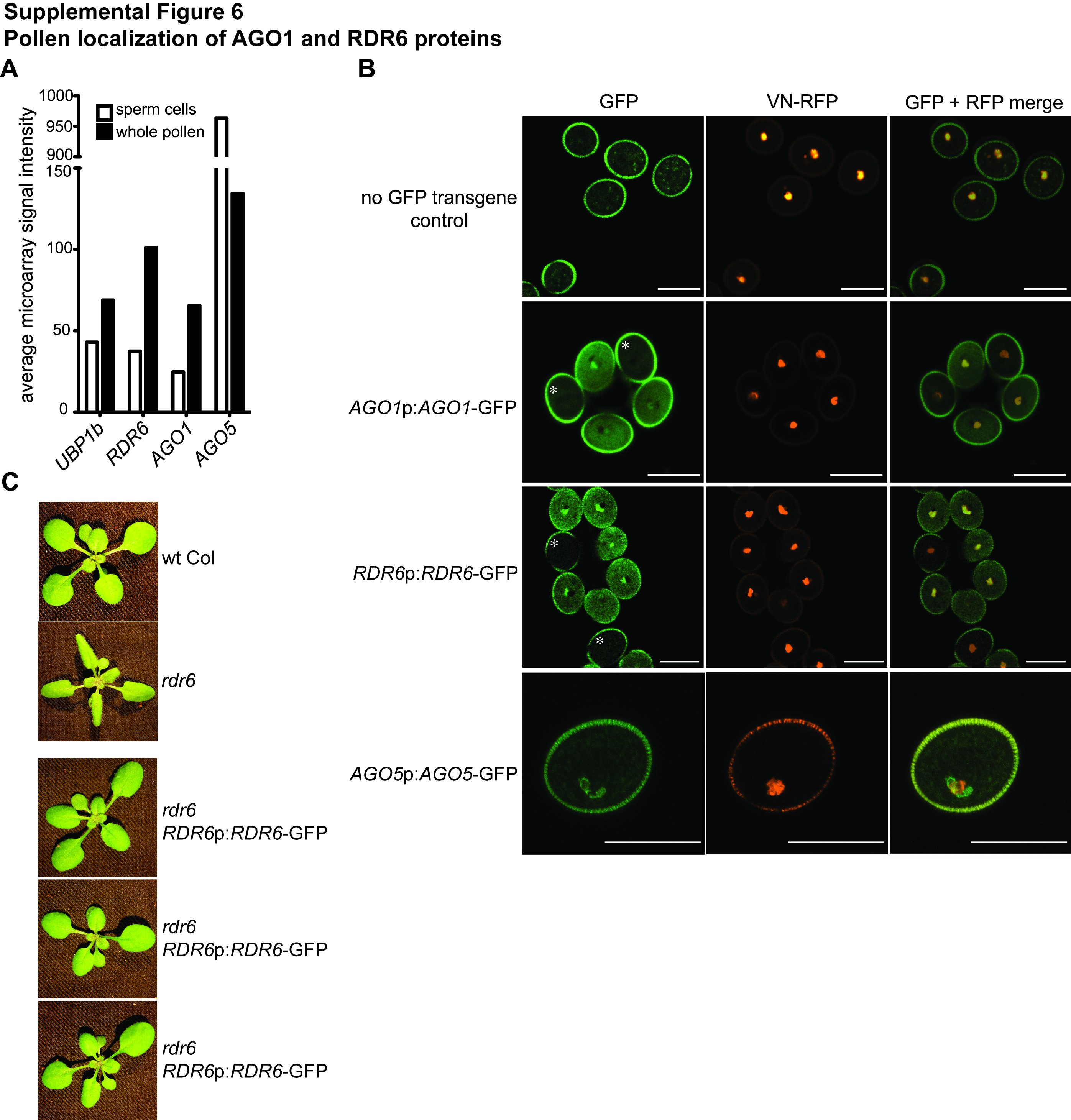

Supplement: Figure S6 — Pollen localization of RDR6 and AGO1. (A) Expression values from microarray data mined from Borges et al. of purified sperm cells and whole mature pollen [45]. UBP1b, RDR6 and AGO1 transcripts are not enriched in sperm cells. AGO5 is shown as a control of a known sperm-specific protein [81]. (B) Fluorescence microscopy images of mature pollen grains expressing GFP fused to the RDR6, AGO1 or AGO5 protein, each under control of their own native promoters. The transgenes were generated by cloning the promoters and open reading frames of the proteins (including introns) into the binary plasmid pMDC107. Transgenes were transformed into a line expressing RFP in the pollen vegetative nucleus (VN-RFP) [82]. Plants hemizygous for the transgene were used for analysis, and pollen grains that did not inherit the transgene are marked with an asterisk. pAGO5:AGO5-GFP is shown as a control for a protein that has known sperm cell localization [81]. In the images of pAGO1:AGO1-GFP and pRDR6:RDR6-GFP, dark shadows of the sperm cells in the vegetative cell cytoplasm can be seen. Scale bars are 20 microns. (C) Complementation of the rdr6 mutant narrow leaf phenotype with the RDR6p:RDR6-GFP transgene from part B. All plants are 14 days old. The pAGO1:AGO1-GFP transgene did not complement the ago1-11 seedling phenotype (data not shown). (TIF) [file pgen.1002474.s006.tif]
